# Telephone Triage Service Data for Detection of Influenza-Like Illness

**DOI:** 10.1371/journal.pone.0005260

**Published:** 2009-04-17

**Authors:** W. Katherine Yih, Kathryn S. Teates, Allyson Abrams, Ken Kleinman, Martin Kulldorff, Robert Pinner, Robert Harmon, Stanley Wang, Richard Platt

**Affiliations:** 1 Department of Ambulatory Care and Prevention, Harvard Medical School and Harvard Pilgrim Health Care, Boston, Massachusetts, United States of America; 2 Influenza Branch, Division of Viral and Rickettsial Diseases, National Center for Infectious Diseases, Centers for Disease Control and Prevention, Atlanta, Georgia, United States of America; 3 Department of Ambulatory Care and Prevention, Harvard Medical School and Harvard Pilgrim Health Care, Boston, Massachusetts, United States of America; 4 Office of Surveillance, National Center for Infectious Diseases, Centers for Disease Control and Prevention, Atlanta, Georgia, United States of America; 5 Optum, a UnitedHealth Group company, Golden Valley, Minnesota, United States of America; U.S. Naval Medical Research Center Detachment/Centers for Disease Control, United States of America

## Abstract

**Background:**

Surveillance for influenza and influenza-like illness (ILI) is important for guiding public health prevention programs to mitigate the morbidity and mortality caused by influenza, including pandemic influenza. Nontraditional sources of data for influenza and ILI surveillance are of interest to public health authorities if their validity can be established.

**Methods/Principal Findings:**

National telephone triage call data were collected through automated means for purposes of syndromic surveillance. For the 17 states with at least 500,000 inhabitants eligible to use the telephone triage services, call volume for respiratory syndrome was compared to CDC weekly number of influenza isolates and percentage of visits to sentinel providers for ILI. The degree to which the call data were correlated with either CDC viral isolates or sentinel provider percentage ILI data was highly variable among states.

**Conclusions:**

Telephone triage data in the U.S. are patchy in coverage and therefore not a reliable source of ILI surveillance data on a national scale. However, in states displaying a higher correlation between the call data and the CDC data, call data may be useful as an adjunct to state-level surveillance data, for example at times when sentinel surveillance is not in operation or in areas where sentinel provider coverage is considered insufficient. Sufficient population coverage, a specific ILI syndrome definition, and the use of a threshold of percentage of calls that are for ILI would likely improve the utility of such data for ILI surveillance purposes.

## Introduction

The principal objective of surveillance for influenza and influenza-like illness (ILI) is to guide public health prevention programs to mitigate the morbidity and mortality caused by annual influenza epidemics, which cause approximately 36,000 deaths each year in the United States [1]. It is hoped that these surveillance systems will also help warn of and track the development of the anticipated next influenza pandemic, which may cause over a million deaths in this country [2]. Influenza surveillance components coordinated nationally by the CDC include: the number and percentage of all outpatient visits to sentinel providers that are for ILI, virologic surveillance for influenza virus type and subtype, mortality data reported through the 122 Cities Mortality Reporting System, pediatric hospitalization rate estimates, nationally notifiable influenza-associated pediatric deaths, and state influenza activity as reported by state and territorial epidemiologists [3].

Less traditional electronic data have been proposed as additional sources of surveillance information, and several authors have shown that ambulatory care and emergency department (ED) data have been useful for identifying ILI activity [4]–[11]. In the United Kingdom and Canada, there have been some assessments of the utility of national or provincial telephone health advice lines as a source of surveillance data for influenza and other communicable diseases [12]–[17]. Using weekly numbers of laboratory reports of the main respiratory pathogens, Cooper et al. [18] created models capable of providing weekly estimates of the proportions of calls due to specific microbiological causes, including influenza. In the U.S. as well, data from national nurse telephone triage services, although lacking laboratory confirmation, might be useful for ILI surveillance for reasons of timeliness and the potential to complement existing surveillance—it is possible that these data can be collected more quickly or efficiently than the weekly reports currently received by CDC, and they may be available in locations or at times during the year when conventional ILI surveillance systems do not operate.

For this analysis we retrospectively compared respiratory illness surveillance using electronically collected data from a national nurse telephone triage service to CDC's national influenza and ILI surveillance data for the 2004–2005 season. (For a variety of reasons, telephone triage service data from subsequent seasons were not available for this analysis.) The 2004–2005 season was characterized as “moderate” by CDC; it was associated principally with influenza A (H3N2) and peaked in February [19]. Our goals were 1) to determine the validity of this non-traditional data source in describing the influenza season, utilizing the CDC data on number of influenza isolates and percentage of sentinel provider ILI visits as the gold standard, and 2) to consider what, if any, advantage could be gained by adding such a data source to existing national ILI surveillance indicators.

## Methods

### Observation period and geographic areas

We examined the period from October 3, 2004 through April 16, 2005 (week 40 of 2004 through week 15 of 2005). We compared CDC data and respiratory illness call data for the 17 states with at least 500,000 residents eligible (by virtue of their health insurance plan) to use the nurse telephone triage service. All but one of CDC's nine influenza surveillance regions were represented—three states were in the Mid-Atlantic region, three in East North Central, two in West North Central, three in South Atlantic, one in East South Central, two in West South Central, two in Mountain, and one in Pacific. The surveillance region with no states with 500,000 residents eligible to use the telephone triage service was New England.

### CDC virologic and sentinel provider percentage ILI data

Two CDC influenza surveillance components were used for this comparison: the number of virologic specimens positive for influenza and the percentage of all sentinel provider visits reported as being for ILI. The absolute number of positive specimens is reported weekly by the states and includes specimens from sentinel providers as well as other sources.

The total number of patients seen for any reason and the number of those patients with ILI, from which is calculated the percentage of patients seen for ILI, are reported on a weekly basis by sentinel providers. ILI is defined as temperature of ≥100°F plus a cough and/or sore throat in the absence of a known cause other than influenza. Nationwide, 2,252 sentinel providers were enrolled in the U.S. Sentinel Provider Network during the 2004–2005 influenza season. Of these, 1,323 reported regularly, having submitted reports for at least 16 (about half) of the weeks during the reporting period of October through May. There were 1,291 sentinel providers enrolled in the 17 states in this study, of which 753 reported regularly. Having at least one regularly reporting sentinel provider per 250,000 population is CDC's goal for state ILI surveillance.

The absolute number of positive specimens from sentinel providers and other sources correlates well with CDC percentage ILI data nationally and regionally (CDC, Influenza Branch, unpublished data).

The CDC viral isolate and percentage ILI data used in this analysis were as of May 11, 2005. The numbers of sentinel providers in 2004–2005 were as of December 1, 2006.

### Telephone triage service data

Optum, a national managed-care nurse telephone triage service with 26 million patients in 50 states eligible to use its services through their managed care plans, was one of several providers of aggregate data on patient diagnoses to the National Bioterrorism Syndromic Surveillance Demonstration Program (NDP) [20], [21]. As part of ongoing syndromic surveillance conducted by the NDP, Optum routinely sent to the NDP's data center next-day daily counts of respiratory illness (as well as other syndromes) for each zip code with at least one caller conforming to the syndrome definition. Calls made to Optum for health information rather than for advice about a current case of illness were coded differently and did not enter the syndromic surveillance system.

**Figure 1:**
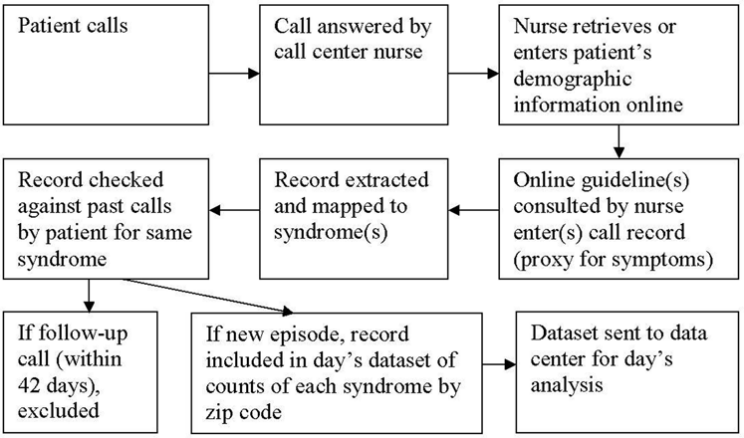
Flow of telephone triage service information, from patient call to analysis.

The flow of information, from the point the patient dialed the phone to receipt of aggregate counts data by the NDP's data center, happened as follows and as shown schematically in Figure 1: Each patient call to Optum was distributed to one of its national call centers according to the availability of personnel to answer, not according to geography. Upon answering a call, the nurse would go online; call up or collect the patient's demographic information, including zip code of residence, in a new call record; and over the course of the conversation consult one or more online “guidelines.” Guidelines were electronic documents about conditions or symptoms, such as “Sore Throat/Adult” or “Influenza/Pediatric.” Whenever a nurse accessed a guideline, the guideline title would automatically be entered into the call record, serving as an indicator of the patient's symptoms. Multiple guidelines could be consulted during a single call, depending on the variety of the patient's symptoms. Each night, patient call records with guideline titles previously determined to be of interest to the NDP for syndromic surveillance purposes were extracted automatically from the Optum data system, in a uniform format specified by the NDP, to a directory accessible to software provided to Optum data-managers by the NDP. The distributed software then mapped patient calls to syndromes (e.g., respiratory), which had been previously defined with CDC collaboration, and then identified which of these represented *new* episodes of illness, ignoring records of patient calls in any syndrome that occurred within 42 days of an earlier call by the patient for the same syndrome. A call could be counted in multiple syndromes; for example, if the two guidelines “Blood in Stools/Pediatric” and “Cough/Pediatric” were accessed, the call would be counted in three syndromes: Gastrointestinal, Hemorrhagic, and Respiratory. A daily file was created containing counts of new episodes of each syndrome by zip code for the preceding day, and this was sent electronically in encrypted form to the NDP's data center. All extraction, processing, and transfer procedures were automated, so no extra manual data entry or active reporting was required of Optum staff.

The 24 Optum guideline titles corresponding to respiratory syndrome are listed in Table 1. This syndrome was defined broadly and, although it included cough, sore throat, and influenza, it included many other symptoms as well.

**Table 1:** Optum guidelines whose utilization by the nurse responding to the call would lead to a classification of respiratory syndrome.

Breathing Difficulty/Severe/Pediatric
Breathing Difficulty/Adult
SARS/Possible/Adult
Cough/Adult
Cough/Pediatric
Sore Throat/Pediatric
Colds/Pediatric
Bronchiolitis/Follow-Up Call/Pediatric
Sore Throat/Adult
Cold or Upper Respiratory Infection/Possible/Adult
Influenza/Pediatric
Respiratory Symptoms/Multiple/Guideline Selection/Pediatric
Asthma Attack/Pediatric
Bluish Skin or Body Part/Pediatric
Chest Pain/Adult
Chest Pain/Pediatric
Congestion/Guideline Selection/Pediatric
Croup/Pediatric
Hoarseness or Laryngitis/Adult
Hoarseness/Pediatric
Sinus Pain and Congestion/Pediatric
Strep Throat Infection/Follow-Up Call/Pediatric
Wheezing/Adult
Wheezing/Other than Asthma/Pediatric

### Analysis

State-level analyses were done for the 17 states with at least 500,000 inhabitants eligible to use the telephone triage services; we reasoned that states with fewer than that number of eligibles would not have robust enough call data to justify analysis. CDC weekly data on number of influenza isolates and percentage of total visits to sentinel providers for ILI were compared to the total number of calls for respiratory syndrome received in the same week. Pairwise correlations among the three data types were calculated, with lag times of 0, 1, 2, 3, and 4 weeks, using the Pearson correlation test. The number of sentinel providers and the number per 250,000 population in those 17 states were also tabulated.

Six states were selected as “representative” for graphical display: the two with the highest correlations between call data and CDC percentage ILI data (California and Wisconsin), the two with the lowest such correlations (Arizona and New Jersey), and two in the middle of the range (Missouri and Ohio). The call data presented in the graphs are rolling seven-day totals of calls for respiratory syndrome, computed day by day for the seven-day period ending on the date in question.

Influenza surveillance coordinators in the six named states were approached for permission to identify their states' data, and all consented. Coordinators in the other 11 states were not asked for such permission; each of those states is represented by a code letter in the tables.

## Results

Introducing lag-times did not increase the correlation coefficients. Correlations among the three data types with no lag are shown in Table 2, along with call service usage and CDC sentinel provider coverage. The correlation between CDC viral isolate and percentage ILI data was highest overall, with a median correlation coefficient of 0.80 (range 0.46–0.97); 12 of the 17 states (Set A: CA, WI, D, E, F, G, MO, J, K, L, M, AZ) had a coefficient of ≥0.75. The next best correlated were the telephone triage data and CDC percentage ILI data, with a median correlation coefficient of 0.74 (range 0.34–0.89) and eight states (Set B: CA, WI, D, E, F, G, H, MO) with a coefficient of ≥0.75. The median correlation between call data and CDC viral isolate data was 0.65 (range 0.35–0.83), and four states (Set C: CA, D, E, MO) had a correlation coefficient of ≥0.75 (and ≥0.80). Set C was a complete subset of both Set B and Set A. Seven of the eight states in Set B were also in Set A.

**Table 2:** Telephone triage service usage, number and coverage of CDC sentinel providers, and correlations among three data types, for states with at least 500,000 eligible to use the telephone triage service.

	Population eligible to use nurse call centers	Number of calls in 2004	Number of calls per eligible population	Number of CDC sentinel providers in 2004–2005	Number of CDC sentinel providers per 250,000 population	Number of CDC sentinel providers reporting regularly	Number of CDC sentinel providers reporting regularly per 250,000 population	Correlation between calls and CDC viral isolates	Correlation between calls and CDC sentinel provider data	Correlation between CDC sentinel provider data and CDC viral isolates
CA	3,136,617	72170	2.30%	166	1.16	71	0.50	0.80	0.89	0.85
WI	825,255	20374	2.47%	110	5.00	76	3.45	0.67	0.87	0.81
State D	558,705	20432	3.66%	32	1.74	28	1.52	0.82	0.86	0.96
State E	2,529,566	56049	2.22%	73	0.81	32	0.36	0.83	0.85	0.97
State F	1,005,895	18924	1.88%	87	1.71	68	1.34	0.68	0.85	0.77
State G	1,871,970	62857	3.36%	120	1.73	86	1.24	0.66	0.84	0.80
State H	1,050,846	75841	7.22%	27	1.32	18	0.88	0.69	0.79	0.46
MO	812,613	20862	2.57%	35	1.52	27	1.17	0.80	0.75	0.77
OH	1,866,557	38970	2.09%	86	1.88	31	0.68	0.60	0.74	0.67
State I	638,423	13140	2.06%	39	1.65	13	0.55	0.37	0.68	0.68
State J	1,908,961	37170	1.95%	123	1.59	62	0.80	0.65	0.66	0.90
State K	1,912,138	6694	0.35%	67	3.72	56	3.11	0.57	0.65	0.81
State L	873,240	51612	5.91%	62	1.81	54	1.58	0.35	0.63	0.78
State M	670,997	33129	4.94%	108	3.03	31	0.87	0.52	0.52	0.92
State N	882,089	15969	1.81%	71	1.43	49	0.99	0.49	0.51	0.66
AZ	748,838	31072	4.15%	54	2.35	43	1.87	0.35	0.51	0.87
NJ	718,395	14305	1.99%	31	0.89	9	0.26	0.64	0.34	0.53
**Median:**								**0.65**	**0.74**	**0.80**

Rows are sorted by correlation between telephone triage calls and percentage of visits for ILI from CDC sentinel providers (penultimate column).

All eight states with at least one regularly reporting sentinel provider per 250,000 population had correlations between the two CDC data types of ≥0.75, i.e. were in Set A; four states with lower sentinel provider coverage also had correlations in that range. But there was no other obvious relationship between the density of sentinel providers and the correlations among any of the three data types.

The ratio of 2004 call volume to the number of people eligible to use the service ranged from 0.35% to 7.22% for the 17 states. There was no clear relationship between this usage proxy and the correlation patterns. For example, states with highest usage did not have the highest correlations between calls and sentinel provider data.

**Figure 2:**
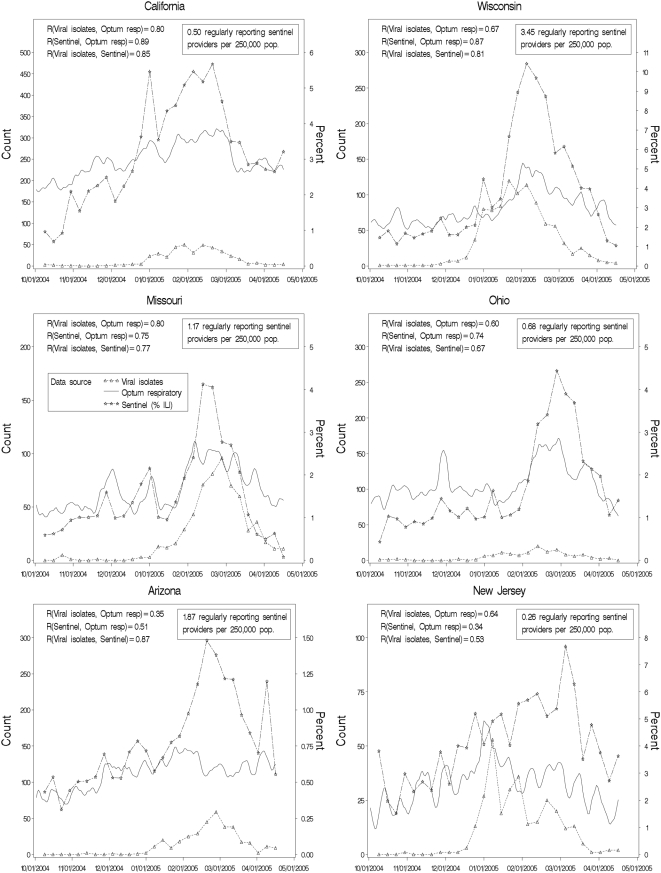
Weekly CDC influenza isolates, CDC percentage ILI from sentinel providers, and Optum respiratory-syndrome call volume. The selected states are the two with the highest correlations between CDC sentinel provider data and call data (California and Wisconsin), the two with the lowest correlations (Arizona and New Jersey), and the two in the middle of the range (Missouri and Ohio).

Values of the three data types over the course of the season are presented graphically for the six “representative” states in Figure 2.

## Discussion

In this study of the potential utility of call data for ILI surveillance, we found considerable variation among states—even ones with adequate sentinel provider coverage—in the degree to which the call data were correlated with either CDC viral isolates or sentinel provider percent ILI data. Thus, the face validity of these call data is by no means uniformly high. There are several possible reasons, including a) insufficient coverage of the population, b) lack of specificity due to too broad a syndrome definition, c) lack of specificity due to the indirect nature of telephone diagnosis, and d) inappropriate evaluation methods. We consider each of these, with possible remedies, in turn.

a) *Coverage*. Population coverage of the telephone triage company described here is uneven and very sparse in many states. Although we limited the analysis to states with at least 500,000 people eligible to use the telephone triage service, it is possible that coverage and use patterns were insufficient or too heterogeneous for the data to accurately reflect ILI morbidity even in those states. A unified system that operates at the national level, like the U.K.'s universal National Health Service, with its integrated NHS Direct call system [12]–[15], could provide sufficient coverage and potentially help elucidate how influenza or ILI moves across the continent [22]. In the U.S, where telephone triage services are not widely used except in conjunction with large managed care organizations [23], they could be useful for surveillance at a more local level.

b) *Syndrome definition*. Our respiratory syndrome definition included many more conditions than ILI, such as asthma, wheezing, hoarseness, and sinus pain, and fever was not a criterion. It seems likely that using an ILI-specific syndrome definition would have improved the performance of the call data relative to the CDC data and increased its “peakedness,” helpful in determining when influenza first causes noticeable morbidity and when it peaks. Although other pathogens causing ILI, such as respiratory syncytial virus, parainfluenza, and adenovirus, also circulate in the winter months, a number of investigators have found a generally close relationship in temporal patterns between laboratory data on influenza and ILI data from clinical sources [22], [24] as well as directly between laboratory data on influenza and ILI (“cold/flu” and fever) data from telephone triage [18].

c) *Telephone diagnosis vs. direct clinical diagnosis*. One might expect an unacceptable loss of specificity in using telephone health data, but several studies have found that call data and clinical data are closely related for ILI and certain other syndromes [14], [17], [18], [25], and it has been argued on the basis of such results that calls are a “timely, useful and representative data stream that shows promise for integration into a real-time syndromic surveillance system” [17].

d) *Evaluation methods*. It is not self-evident that the degree of correlation with the CDC surveillance data is the best measure of utility of the telephone triage data for ILI surveillance. Other comparative approaches have been used or suggested (especially to compare timeliness of data sources), including cross-correlation time series modeling [23], [24], comparison of peaks [22], and comparison of aberration detection [26]. On reviewing the literature on influenza surveillance, Dailey et al. [26] concluded that an aberration detection method (e.g., use of a threshold, CUSUM, or scan statistic) was preferable to both comparison of peaks and correlation. In previous unpublished work, we have not found the application of scan statistics to syndromic ILI data to be informative. However, a method in which a threshold for percentage of encounters for ILI is established is appealing; Cooper et al. [18] demonstrated that age-group-specific thresholds of percentage of total calls that are for “cold/flu” syndrome, derived by means of Poisson regression models, were useful, providing 6–14 days of advance warning of seasonal influenza activity.

In synthesis, in the absence of a universal health care system, telephone triage data, even when from a single company with national scope, will be patchy in coverage and therefore not a reliable source of ILI surveillance data on a national scale. However, in states with higher correlation between the call data and CDC sentinel surveillance percentage ILI and/or virologic data (and a sizeable number of residents eligible to use a telephone triage service), call data may serve as a useful adjunct to CDC influenza/ILI surveillance data, for example at times when sentinel surveillance is not in operation or in areas where sentinel provider coverage is considered insufficient. Utility for ILI surveillance is likely to be higher if an ILI-specific syndrome definition can be employed in the electronic data collection. A threshold method of aberration detection seems most promising for near-real-time ILI surveillance. The relative advantage to public health of analyzing telephone triage data as a separate, potentially more timely data stream vs. combining these data with ED or ambulatory care data for a larger, possibly more robust source remains to be determined.
